# A Population of M2 Macrophages Associated With Bone Formation

**DOI:** 10.3389/fimmu.2021.686769

**Published:** 2021-10-12

**Authors:** Elizabeth Olmsted-Davis, Julio Mejia, Elizabeth Salisbury, Zbigniew Gugala, Alan R. Davis

**Affiliations:** ^1^ Center for Cell and Gene Therapy, Baylor College of Medicine, Texas Children’s Hospital and Houston Methodist Hospital, Houston, TX, United States; ^2^ Department of Pediatrics – Section Hematology/Oncology, Baylor College of Medicine, Houston, TX, United States; ^3^ Department of Orthopedic Surgery, Baylor College of Medicine, Houston, TX, United States; ^4^ Department of Orthopedic Surgery and Rehabilitation, University of Texas Medical Branch, Galveston, TX, United States

**Keywords:** heterotopic ossification, brown-fat-like macrophages, M2 macrophages, macrophage function, single-cell RNAseq

## Abstract

We previously identified transient brown adipocyte-like cells associated with heterotopic ossification (HO). These ancillary cells support new vessel synthesis essential to bone formation. Recent studies have shown that the M2 macrophage contributes to tissue regeneration in a similar way. To further define the phenotype of these brown adipocyte-like cells they were isolated and characterized by single-cell RNAseq (scRNAseq). Analysis of the transcriptome and the presence of surface markers specific for macrophages suggest that these cells are M2 macrophages. To validate these findings, clodronate liposomes were delivered to the tissues during HO, and the results showed both a significant reduction in these macrophages as well as bone formation. These cells were isolated and shown in culture to polarize towards either M1 or M2 similar to other macrophages. To confirm that these are M2 macrophages, mice received lipopolysacheride (LPS), which induces proinflammation and M1 macrophages. The results showed a significant decrease in this specific population and bone formation, suggesting an essential role for M2 macrophages in the production of bone. To determine if these macrophages are specific to HO, we isolated these cells using fluorescence-activated cell sorting (FACS) from a bone defect model and subjected them to scRNAseq. Surprisingly, the macrophage populations overlapped between the two groups (HO-derived *versus* callus) suggesting that they may be essential ancillary cells for bone formation in general and not selective to HO. Of further note, their unique metabolism and lipogenic properties suggest the potential for unique cross talk between these cells and the newly forming bone.

## Introduction

Although the focus has been placed on isolating stem cells for regenerative medicine, recent studies suggest an essential role for microenvironmental factors such as nutrients, inflammatory signaling, and growth factors/cytokines/adipokines (1). One of the best studied cells that regulate the microenvironment is the macrophage that has significant plasticity to alter its function and phenotype depending on the environment. Thus, they can be lipogenic (2), angiogenic (3), immune suppressive (4) or support expansion of adaptive immunity through secretion of proinflammatory factors (5). In our own studies of *de novo* bone formation, referred to as heterotopic ossification (HO), we noted the presence of a brown adipocyte-like cell (BAT), which is highly transient and has a unique uncoupled metabolism (6–8). This cell appears rapidly in mice (8) and in humans (7) during the early stages of HO. We have found that this BAT-like cell is also critical for eliciting rapid changes in the nutrient and oxygen microenvironment (6). These transient brown adipocytes also express uncoupling proteins 1 and 2 (Ucp1 and Ucp2), which “uncouple” oxidative phosphorylation from the production of ATP. While this does not stop the production of ATP, it does reduce the buildup of protons and has been shown to prevent the production of reactive oxygen species. The outcome of this process is the generation of heat, and increased water, which is pumped out of the cells and may appear as edema. Studies have shown that these cells possess elevated mitochondria, exhibit robust, but uncoupled, aerobic metabolism, and can effectively induce localized regions of hypoxia required for chondrogenesis (6), which, in turn, stimulates the hypoxia inducible factor (Hif) pathway and ultimately angiogenesis (9). These cells have also been shown to secrete vascular endothelial growth factor A (Vegfa) (9) and produce hypoxia inducible factor 1(Hif1) (6), which are associated with robust angiogenesis (9).

While the phenotype of these BAT-like cells appeared to be adipogenic, recent studies in tissue regeneration suggest that M2 macrophages can have increased mitochondrial expression (2), regulate metabolism and that these processes appear to support angiogenesis (3), cell proliferation (2), and suppress proinflammation (3), since the pro-inflammatory (M1) processes may be destructive rather than constructive. Several studies have reported that M2 macrophages, beyond expressing a number of adipogenic proteins, including peroxisome proliferator-activator receptor gamma (PPARɣ), also have elevated mitochondria and small lipid vacuoles (10). Thus, in the studies presented here, we performed scRNAseq on these BAT-like cells isolated from HO to determine their phenotype and relationship to M2 macrophages.

The studies presented here describe a new set of M2-like macrophages associated with HO. Transcriptome comparisons suggest these cells are strikingly similar to border-associated macrophages (BAMs), recently found at various tissue barriers in the brain (11), suggesting a new category of M2 macrophages may exist to support tissue regeneration and repair. Surprisingly, by transcriptome comparison, similar macrophages were identified in fracture callus, suggesting these M2-like macrophages may contribute a common function to different types of bone formation/tissue regeneration.

## Materials and Methods

### Cell Culture

Primary bone-marrow-derived macrophages were prepared from whole bone marrow isolated from Colony stimulating factor receptor-green fluorescent protein (Csfr1-GFP) mice. Briefly, whole bone marrow was suspended in phosphate-buffered saline and passed through a 40 µm cell strainer. Cells were then propagated in Dulbecco’s modified Eagle’s medium (DMEM) supplemented with 10% fetal bovine serum (FBS), 1X antibiotic-antimycotic, and 40 ng/ml Csf1 (Biolegend 576404, San Diego, CA) in sterile petri dishes in a humidified incubator at 37°C/5% CO_2_. Their phenotype was confirmed using flow cytometry for GFP and found to be >95% pure. Cells were discarded after 20 passages. An immortalized microglial cell line was purchased from the American Type Culture Collection (ATCC) (C8-B4, Manassas, VA). These cells were maintained at 37°C/5% CO_2_ in DMEM supplemented with 10% FBS and 1X antibiotic-antimycotic. ADRβ3^+^ macrophages, isolated by flow cytometry from tissues surrounding HO, were propagated in DMEM supplemented with 10% FBS, 1X antibiotic-antimycotic, and 40 ng/ml Csf1 (Biolegend). Approximately 10-20% of the cells were able to adhere to the culture dish. Cellular adherence was not improved with the use of petri or gelatin-coated dishes.

### Polarization Assay

Macrophages (microglia, bone-marrow-derived macrophages, and ADRβ3^+^ macrophages) were plated at a density of 10,000 cells/cm^2^ in culture media (DMEM supplemented with 10% FBS, 1X antibiotic-antimycotic, and 40 ng/ml Csf1). Subsets were placed in similar media containing 20 ng/ml Interleukin 4 (Il4) (M2 polarization) or 200 ng/ml LPS (M1 polarization) and placed at 37°C for 48 hours. The medium was removed, and the cells were washed using phosphate buffered saline. Cells were then lysed using TRIzol Reagent (Life Technologies, Carlsbad, CA) and RNA isolated in accordance with the manufacturer’s instructions. RNA was quantified using a nanodrop machine.

### *In Vivo* Polarization and Lipolysaccharide

An M1-macrophage phenotype was promoted *in vivo* with use of LPS (tlrl-prslps; InvivoGen, San Diego, CA). LPS in saline (50 µl of 0.5 ng/g w/w) was injected into each quadriceps. Saline (50 µl) was used as control. Injections of LPS were performed: 72 hours prior to injection of Bmp2-producing cells and immediately following the injection of Bmp2-producing cells with re-injection every 72 hours. Tissues were analyzed using flow-cytometry and microCT.

### Q-RT-PCR

RNA (5µg) was converted to cDNA using the RT2 First-Strand Kit containing SYBR green (Qiagen, Germantown, MD). Real-time quantitative polymerase chain reaction (qPCR) analysis was done using the RT2qPCR Primer Assay (Qiagen, Germantown, MD). For normalization, Tbp (TATA box binding protein; catalog no. PPM03560E-200, Qiagen) was found to be the best internal control. The cDNA was subjected to quantitative real-time reverse transcriptase PCR analyses in parallel using a 7900HT PRISM Real-Time PCR machine and SDS 2.3 software (Applied Biosystems, Carlsbad, CA). The Ct values, where Ct is defined as the threshold cycle of PCR at which cDNA reaches exponential amplification, were determined for each biological sample in duplicate. Values were normalized against Tbp and expressed relative to RNA isolated from control tissues. Relative gene expression was determined using ΔΔCt method, and the means and SEMs were calculated.

### Primers

Mrc1F: 5’-CGGAATTTCTGGGATTCAGCTTC-3’; Mrc1R: 5’-CTCTGTTCAGCTATTGGACGC-3’;
Arg1F: 5’-CTCCAAGCCAAAGTCCTTAGAG-3’; Arg1R: 5’-AGGAGCTGTCATTAGGGACATC-3’;
Nos2F: 5’-GTTCTCAGCCCAACAATACAAGA-3’; Nos2R: 5’GTGGACGGGTCGATGTCAC-3’;
Tnfα F: 5’-GACGTGGAACTGGCAGAAGAG-3’; TnfαR: 5’-TTGGTGGTTTGTGAGTGTGAG-3’

### Flow Cytometry

The cells from hindlimb tissues were isolated and digested with collagenase type 2 as previously described (8). Briefly, hindlimb muscle tissue was dissected from the skeletal bone into cold Hank’s buffered saline solution (HBSS) and dissociated by mincing the tissues and incubating for 45 minutes at 37°C in 0.2% type 2 collagenase (Worthington) in HBSS. An equal volume of DMEM supplemented with 10% fetal bovine serum was added to quench the digestion reaction. Dissociated cells were centrifuged, triturated, filtered through nylon mesh, and resuspended in cell staining buffer. FACS was performed using a FACS Aria II cell sorter (BD Biosciences, San Jose, CA) equipped with analyzing software (BD FACSDiva software version 8.0.1, BD Biosciences). Cells were incubated with antibodies (ADRβ3 antibody; ab59685, chicken polyclonal; 1:250 (Abcam, Cambridge, MA). Ccr2 antibody (ab203128, rabbit monoclonal, 1:200, Abcam, Cambridge, MA). Cd11b-SB780 conjugated antibody (78-0112-82, rat monoclonal, 1:100, Invitrogen Life Technologies, Carlsbad, CA). Cd68 antibody (MCA1957GA, rat monoclonal; 1:100; Bio-Rad, Hercules, CA). Cx3cr1 antibody (ab8021, rabbit polyclonal; 1:100, Abcam, Cambridge, MA). F4/80 antibody (MCA497A647, rat monoclonal; 1:100, Bio- Rad, Hercules, CA). Mrc1 antibody (ab64693, Rabbit polyclonal; 1:200; Abcam, Cambridge, MA). Trem2 antibody (rabbit polyclonal, bs-2723r; 1:100, Bioss, Boston, MA). Tmem119 antibody (rabbit monoclonal, ab210405; 1:100, Abcam, Cambridge, MA) and then visualized using Alexa Fluor secondary antibodies (1:500 dilution; 488, 594, or 647; Invitrogen Life Technologies, Carlsbad, CA). For cell sorting, labeled cells were separated based on their fluorescence intensity and all populations collected with 95% purity in tubes pre-coated with bovine serum albumin and containing FBS.

### Macrophage Ablation Assay

Clodronate liposomes were used to selectively ablate macrophage populations. Clodronate and control liposomes (CP-005-005) were purchased from Sapphire North America (Ann Arbor, MI). To ablate both local and systemic macrophage activity 50 µL of liposome was injected into each quadriceps of the animal and 100 µL of liposome was injected into the intraperitoneal cavity. Injections of liposome were performed: 72 hours prior to injection of BMP2-producing cells and immediately following the injection of BMP2-producing cells with re-injection every 72 hours. Tissues were analyzed using flow-cytometry and microCT.

### Ucp1Cre:Csf1r^floxSTOPflox^ Diphtheria Toxin Receptor-mcherry Mice

The Ucp1Cre:Csf1r^floxSTOPflox^DTR-mcherry mouse was constructed by crossing the Ucp1Cre mouse (Jackson Laboratories Stock No: 024670) with the Csfr1 DTRmcherry mouse (Jackson Laboratories Stock No: 024046). Offspring then possessed mCherry in cells expressing both Csf1r and Ucp1. HO was induced in the offspring through delivery of AdBMP2-transduced cells, and the resultant bone formation was assessed for mCherry^+^ ADRβ3^+^ macrophages. The results showed 94.8 +/- 0.45% of the ADRβ3+ cells were labeled with mCherry. Mice serving as a control were then given intraperitoneal injections of diphtheria toxin A every 48 hours starting at the time of BMP2 delivery throughout the experiment and we noted the mCherry+ cells were completely eliminated.

### Microscopy

Tissues were isolated and placed in formalin. The tissues were then decalcified, paraffin embedded, and sectioned serially at a depth of 2-4 μ. Paraffin was removed using xylene and tissues rehydrated and immunostained, for instance, using anti-Ucp1 (Novus Biologicals, Centennial, CO, NB100-2828, goat polyclonal, 1:100 dilution) and anti-Mrc1 (Abcam, b64693, rabbit polyclonal, 1:200 dilution) antibodies. Secondary antibodies were linked with Alexafluor 488 and 597 dyes. Tissues were counterstained and covered with ProLong Glass Antifade Mountant with NucBlue (Invitrogen, P36985). Stained tissue sections were examined using an Olympus BX41 microscope (Olympus Corporation of the Americas, Waltham, MA) equipped with a reflected fluorescence system or by confocal microscopy (LSM 510 META, Zeiss, Inc., Thornwood, NY, USA) using a 20X/0.75NA objective lens. To ensure signal specificity, controls were performed and the specific absorption spectrum from each primary-secondary pair was captured. To locate the tissue regions, every 5th slide was stained with hematoxylin (Harris Hematoxylin, American Mastertech, Lodi, CA) and eosin (Eosin Y Phyloxine B solution, Electron Microscopy Sciences, Hatfield, PA). Hematoxylin and eosin images were captured by bright-field microscopy using the Olympus BX41 microscope.

### BMP2-Induced Heterotopic Bone Formation

Replication-defective early regions 1 and 3 (E1-E3)-deleted human type 5 adenovirus possessing cDNA for Bmp2 (AdBmp2) in region E1 was constructed as previously described (12). Mouse skin fibroblasts were transduced at 5,000 virus particles per cell with 0.75% GeneJammer to achieve greater than 90% transduction efficiency as described previously (13). All adenoviruses vectors were negative for replication competent adenovirus. AdBMP2-transduced cells (5 × 10^6^ cells) were resuspended in saline and injected into the hindlimb muscles. Cells (5 x 10^6^) were confirmed to express approximately 20 ng of BMP2.

### Bone Repair Model

C57BL/6 mice (4-6 months of age) received a unicortical drill defect in the femur diaphysis to induce bone healing. Mice received analgesia prior to surgery and throughout the duration of the experiment in accordance with the Baylor College of Medicine (BCM) Institutional Animal Care and Use Committee (IACUC) approved protocol. Mice were anaesthetized and prepared for surgery. Then under sterile conditions, a lateral 1 cm skin incision was made to expose the femur where the small drill defect (0.7-mm diameter; 1-mm depth) was created in one cortex. Tissues were closed using wound clips and the mice allowed to recover. After 4 days, mice were euthanized, callus tissues isolated and dissociated cells subjected to flow cytometry.

### MicroCT Analysis

Formalin-fixed tissues were scanned at 9.3 μm spatial resolution (SkyScan 1174; Micro Photonics Inc, Allentown, PA). Regions of interest (ROIs) were defined for each specimen to isolate the new mineralized tissue formed within the skeletal musculature at the injection site. The volume of heterotopic bone was calculated using the CTAn software package (Micro Photonics Inc, Allentown, PA) with a density threshold set at 0.2 g/cm^3^. The bone mineral density of the heterotopic bone was assessed following hydroxyapatite phantoms to establish the attenuation coefficients at 0.25 and 0.75 densities. The 3D microCT reconstructions were created using the CTVox software package (Micro Photonics Inc, Allentown, PA). The femur bone defect healing was assessed using the microCT protocol. The volume and mineral densities of the callus within and beyond the confines of the cortical defect were calculated and compared.

### Single-Cell RNAseq

Approximately 5,000 ADRβ3+ cells from either HO or fracture callus were resuspended at 500 cells/ml and provided to the BCM Single Cell Genomics core facility at our institution. An estimated 3,000 cells were successfully processed and coded using a 10X Genomics platform (10X Genomics, Pleasanton, CA) and both libraries were prepared according to the manufacturer’s instructions (10X Genomics). The libraries were sequenced to a depth of 143,000 reads per cell (Genewiz, Summerfield, NJ). Data was processed through a Cell Ranger pipeline (10X Genomics), converted to a sparse matrix and then clustered using the Seurat (version 3) algorithm. The Seurat version 3 algorithm (14) is able to easily separate cell types because it utilizes the mathematically-derived Uniform Manifold Approximation and Projection (UMAP) rather than t-SNE as the dimension reduction technique for machine learning (15). All data were processed using the R and R Studio software packages (Boston, MA).

### Statistical Analysis

A one-way analysis of variance (ANOVA) with a Tukey post-hoc correction for multiple comparisons with a 95% confidence interval (p<0.05) was used for comparisons between treatment groups.

## Results

### Transient Brown Adipocytes [ADRβ3^+^] Are M2-Like Macrophages

**Figure 1:**
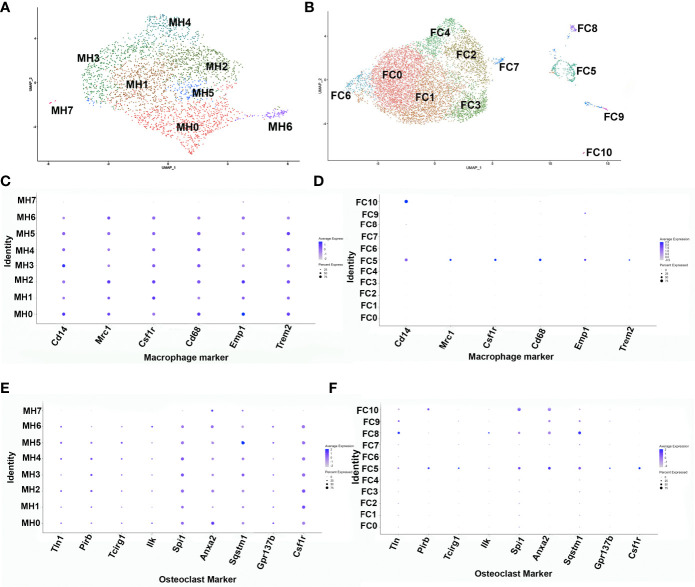
Single-cell transcriptome analysis of ADRβ3+ cells isolated from regions of active bone formation. Analysis of single-cell RNAseq transcriptomes using the Seurat (v3) algorithm. This analysis was done several times with similar results. Transcriptomes associated with the ADRβ3+ cells isolated from **(A)** HO and **(B)** fracture callus. DotPlots of macrophage transcript expression using the Seurat algorithm in **(C)** HO and **(D)** fracture callus. DotPlot depiction of osteoclast-associated transcript expression in **(E)** HO and **(F)** fracture repair.

To further ascertain the phenotype of the transient brown adipocyte-like cells, ADRβ3+ cells were isolated from HO at the stage when they are most abundant. To determine this stage, the kinetics of ADRβ3+ cells was determined by flow cytometry (**Supplemental Figure 2A**). ADRβ3+ cells were then isolated by FACS at 4 days after initial induction or when they are most abundant (**Supplemental Figure 2A**) and subjected to single cell RNAseq. The sequencing data was analyzed using the Seurat (v3) algorithm, which clustered the resultant cells into 8 groups (**Figure 1A**). All clusters, with the exception of MH7, express well-known macrophage-specific transcripts (**Figure 1C**) including Monocyte differentiation antigen (Cd14) (16), Macrophage mannose receptor 1 (Mrc1) (17), Csf1r (18), Protein tyrosine phosphatase RC (Cd45), and Macrophage antigen (Cd68) (19). The fracture callus had far less macrophage markers and only cluster FC5 had a full complement of these markers (**Figure 1D**). It was determined that exosomes can have important downstream effects (20), both positive and negative, and this will be the topic of another study. We show the localization of Adrb3 (red) as well as Cd14-expressing cells (**Supplemental Figure 1**). Note that many of the Adrb3-expressing cells are in the perineurium of peripheral nerves, a localization we have observed previously (8). In addition, we see Cd14-expressing cells in both the endoneurium and perineurium of the nerve. Cd14 is one of the antigens found on the surface of newly differentiated pluripotent stem cells of the myeloid lineage. Cd14 is present as both a glycosylphophatidylinositol-anchored membrane protein (mCd14) and a monocyte-derived soluble protein (sCd14). Both are important for lipopolysaccharide (LPS)-mediated signal transduction. Several clusters express osteoclast-associated transcripts (**Figure 1E**). **Figure 1F** shows that only cluster FC5 has all of the osteoclast transcripts listed. Cluster MH7 has transcripts that are markers of hemogenic endothelial cells Cadherin 5 (Cdh5) (21), Kinase insert domain receptor (Kdr;Flk1) (22), Kit ligand (Kitl) (23), Sry-Box 7 (Sox7) (22), and Sry-Box 17 (Sox17) (24). A list of the top 50 transcripts in each cluster: MH0-MH7 and FC0-FC10 is given in **Supplemental Table 1**. Analysis of cluster MH6 using Pathway Studio (Elsevier, Amsterdam) shows these cells are highly replicating (36%, **Supplemental Figure 3**) suggesting that they may be among the earliest ADRβ3+ cells in HO.

### ADRβ3+ Macrophages Possess a Similar Transcriptome to Border-Associated Macrophages and Macrophages From Peripheral Nerves

Comparison of the transcriptome of ADRβ3+ macrophages and published transcriptomes of other macrophages revealed a striking resemblance to boundary-associated macrophages (BAMs) (11) (**Supplemental Figure 5A**). These macrophages are found in border regions of the brain and while they differ from microglia their function is unknown. Additionally, Wang et al. (25) recently reported the transcriptome of tissue resident macrophages within peripheral nerves, which are very similar to both BAM cells and these ADRβ3+ macrophages. Violin plots were drawn using the published peripheral nerve transcriptome and our own macrophage transcriptome to compare the top 30 transcripts from each population (**Supplementary Figure 5B**). The results show a strong similarity, suggesting these cells may be similar or the same macrophages, suggesting that the ADRβ3+ macrophages are a tissue resident population.

### The ADRβ3+ Macrophages Express Adipocyte-Associated Transcripts

**Figure 2:**
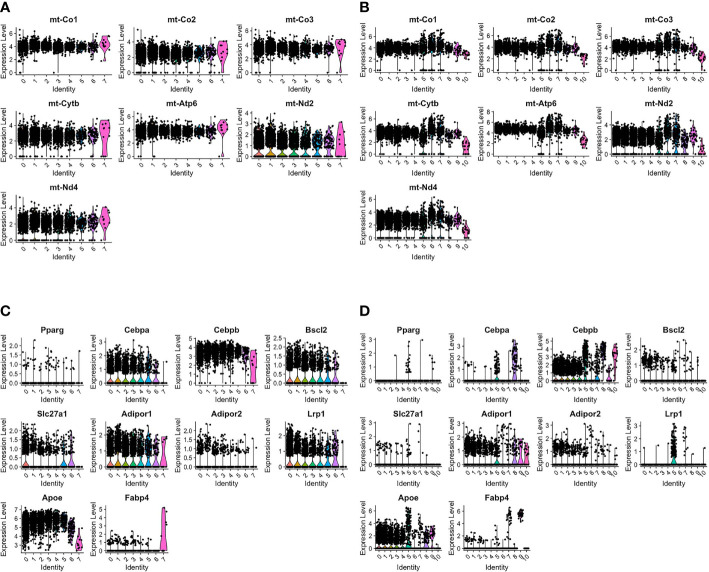
ADRβ3+ cells express transcripts associated with lipid regulation and have elevated mitochondria. Violin plots depict expression of **(A, B)** mitochondrial transcripts and **(C, D)** adipocyte-associated transcripts in each cluster in ADRβ3+ cells isolated from **(A, C)** HO and **(B, D)** fracture callus.

Previous studies in our laboratory have shown that transient brown adipocytes express adipocyte-associated proteins and have elevated mitochondria (6–8). AdRb3+ macrophages during HO and fracture repair are enriched with mitochondrial transcripts (Figures 2A, B). Analysis of the transcriptome during HO and fracture repair shows expression of many transcripts associated with adipocytes (**Figures 2C, D** respectively). This includes the lipogenic transcripts CCAAT/enhancer binding protein beta (Cepbp) (26), Adiponectin receptor protein 1 (Adipor1) (27), Apolipoproein E (Apoe) (28), Uncoupling protein 2 (Ucp2) (29–31), Atp5b (32), Dead-box helicase 5 (Ddx5) (33), Transforming growth factor beta 1 (Tgfb1) (34), and Lamin A/C (Lmna) (35), which are elevated in all clusters with the exception of the hemangioblast-like cluster, as well as Perlipin 2 (Plin2) (36), and PPAR*γ* (37). 

### ADRβ3^+^ Cells Express Macrophage Markers, but do Not Express Integrin, Alpha-M (CD11b) and Adgre1 (F4/80) on Their Surface

**Figure 3:**
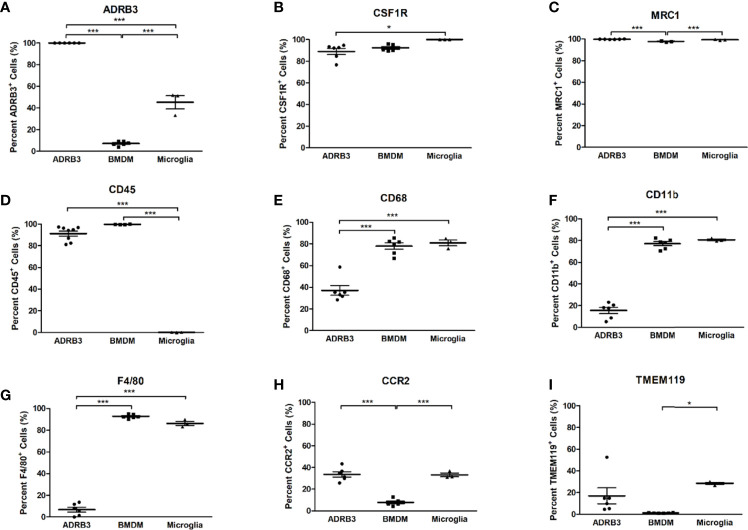
ADRβ3+ cells express several macrophage markers, but lack expression of CD11b and F4/80. Macrophage surface markers were quantified on ADRβ3+ macrophages, bone-marrow-derived macrophages(BMDM), and microglia. Comparisons were made between these cell types to determine commonality. Significant differences (p<0.5) were calculated using an ANOVA with Tukey correction. Symbols represent sample data points, and horizontal lines depict the mean. Standard error of the mean (SEM) is shown by the capped vertical line. *p < 0.05, ***p < 0.001. Flow cytometry was used to quantitate all types of macrophages in both the **Figure 3** legend as well as in the test in the Results. Adrb3+ macrophages, BMDM, and microglia were compared for percent positivity to **(A)** Adrb3; **(B)** Csf1r; **(C)** Mrc1; **(D)** Cd45; **(E)** Cd68; **(F)** Cd11b; **(G)** F4/80; **(H)** Ccr2; and **(I)**, Tmem119.

We next analyzed ADRβ3+ cells for expression of common macrophage surface markers and compared their expression levels to those obtained for bone-marrow-derived macrophages (BMDMs) and microglia, two well characterized macrophage populations (**Figure 3**). Greater than 80% of ADRβ3+ macrophages express Csfr1, Mrc1, and Cd45 (**Figures 3B–D**). Alternatively, less than 15% of the ADRβ3+ macrophages expressed the common macrophage markers Cd11b, and F4/80; whereas greater than 80% of the BMDMs and microglia, were positive for these markers (**Figures 3F, G**).

Less than 40% of ADRβ3+ macrophages expressed Cd68, while approximately 80% of BMDMs and microglia expressed Cd68 (**Figure 3E**). Other markers such as Chemokine-receptor type 2 (Ccr2) and Transmembrane protein 119 (Tmem119) were expressed in 20-30% of the ADRβ3+ macrophages and microglia, but were significantly lower in BMDMs, with <5% of cells expressing these markers (**Figures 3H, I**). For complete comparison, ADRβ3 expression was quantified in the two control populations and it was found that approximately 5% of BMDMs and 45% of microglia expressed this marker (**Figure 3A**).

### ADRβ3+ Macrophages Associated With HO Are Phagocytic and Show Sensitivity to the Clodronate Liposomes

**Figure 4:**
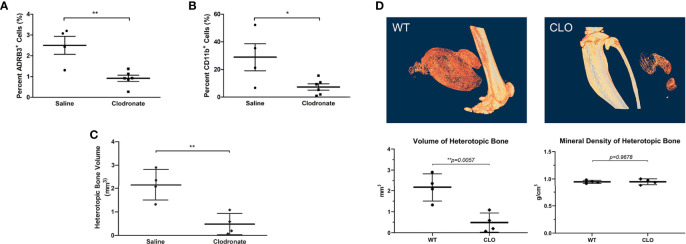
ADRβ3+ macrophages show sensitivity to clodronate liposomes. **(A)** Quantitation of ADRβ3+ cells or **(B)** Cd11b+ macrophages by flow cytometry in tissues isolated 4 days after induction of HO in mice in the presence of clodronate or saline liposomes. **(C)** Analysis of bone volume in tissues isolated 2 weeks after induction of HO in mice in the presence of clodronate or saline liposomes using microCT. Significant differences (p<0.5) were calculated using an ANOVA with Tukey correction. Symbols represent sample data points, and horizontal lines depict the mean. Standard error of the mean (SEM) is shown by the capped vertical line. *p < 0.05, **p < 0.01. **(D)** Individual slices of both saline- and clodronate-treated samples were compared. Also shown for comparison is the mineral density of heterotopic bone.

To determine if the ADRβ3+ cells can function as macrophages by phagocytizing foreign materials in their microenvironment, we injected clodronate or saline liposomes into mice undergoing HO. Clodronate liposomes when ingested are toxic to macrophages (38). We compared whether this macrophage-specific toxin could selectively deplete these ADRβ3+ cells. As a control, to identify any toxicity associated with the liposomes, a subset of mice received saline liposomes rather than drug. The results showed a significant decrease in ADRβ3+ macrophages in mice treated with clodronate liposomes as compared to those given saline liposomes (**Figure 4A**). This suggests that the cells were able to phagocytize the liposomes and had similar sensitivity to clodronate as other macrophages. In the same samples, we also measured the sensitively of Cd11b+ macrophages to treatment with clodronate liposomes (**Figure 4B**). Like the ADRβ3+ macrophages, the Cd11b+ macrophages also decreased, and the resultant bone formation declined significantly (**Figure 4C**) as well, suggesting that macrophages in general may be essential for bone formation. **Figure 4D** shows the decline in bone formation at the resolution of a single-slice along with the overall decline in bone formation with a lack of change in mineral content.

### ADRβ3+ Macrophages Show Plasticity Similar to Other Macrophages

**Figure 5:**
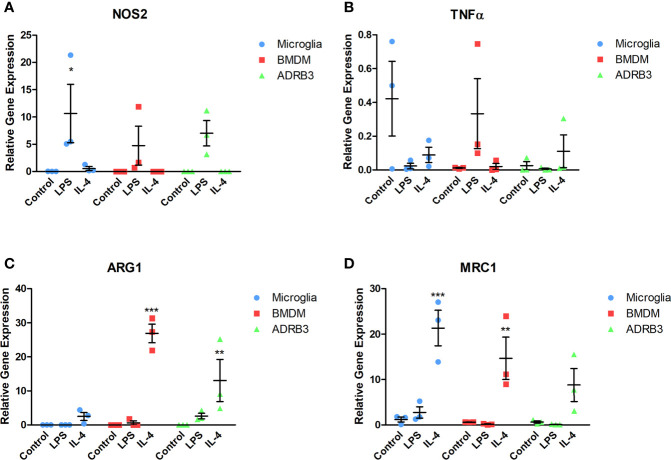
ADRβ3+ cells undergo M1-M2 polarization through response to cytokines in their environment. ADRβ3+ cells, BMDMs, or microglia were isolated and placed in culture in the presence of Il4, LPS, or saline. RNA was isolated 48 hours later, and qRT-PCR was used to quantify genes associated with M1 macrophages **(A)** Inos, **(B)** Tnfα, and M2 macrophages **(C)** Arg1, **(D)** Mrc1. Significant differences (p<0.5) were calculated using an ANOVA with Tukey correction. Bars represent the mean of six samples per group. Standard error of the mean (SEM) is shown by the capped vertical line. *p < 0.05, **p < 0.01, ***p < 0.001.

Macrophages are unique in that they respond to stimuli in their environment by altering their phenotype. To determine if the ADRβ3+ macrophages can undergo polarization, cells were isolated and exposed to either pathogen-derived LPS or Il4 (39) for 48 hours and changes in gene expression were detected by q-RT-PCR for select M1 and M2 transcripts (**Figure 5**). For comparison, microglia and BMDMs were included since they have previously been shown to functionally undergo M1-M2 polarization. Nos2 (iNos), an M1 macrophage marker, was elevated in all macrophage populations after exposure to LPS (**Figure 5A**). Alternatively, Tnfα, another M1 macrophage marker, was elevated in BMDMs after exposure to LPS, but neither microglia nor ADRβ3+ macrophages showed a similar trend. In fact, ADRβ3+ cells appeared to have more Tnfα after exposure to Il4. Tnfα has been suggested to also be expressed in M2 macrophages (40). Alternatively, unstimulated microglia appeared to express Tnfα, but there was high variability between the samples (**Figure 5B**). All macrophage populations appeared to respond to Il 4 by upregulating Arg1 and Mrc1 transcripts, suggesting that they were polarized to an M2 phenotype (**Figures 5C, D**).

### *In Vivo* Analysis of ADRβ3+ Macrophage Plasticity to the M1 Activator LPS

**Figure 6:**
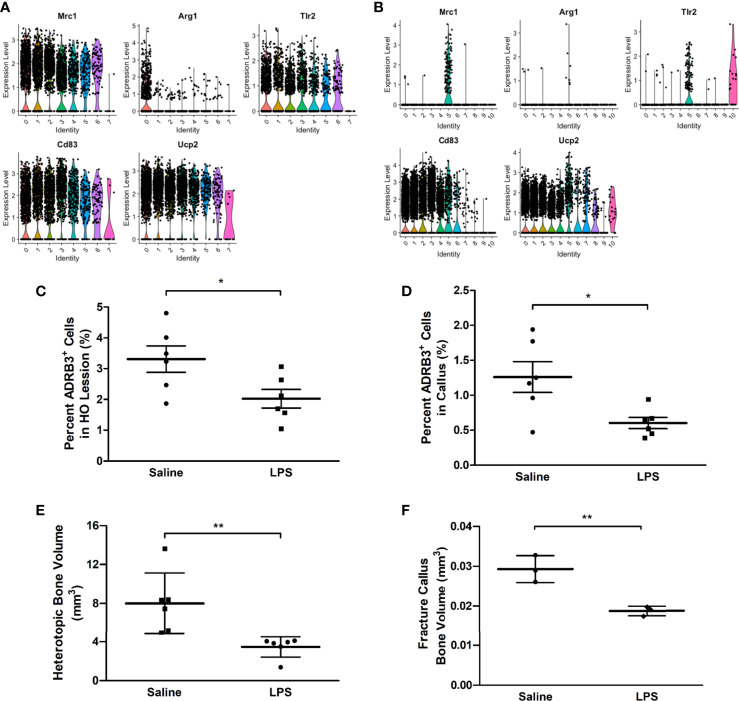
ADRβ3+ macrophages appear to be M2-like and are reduced in number when inflammation is induced in mice using LPS. Transcriptome analysis of the ADRβ3+ macrophages isolated from **(A)** HO and **(B)** fracture callus show a significant number of genes selectively associated with M2 macrophages. ADRβ3+cells were quantified from tissues undergoing **(C)** HO or **(D)** bone injury in mice undergoing a systemic inflammatory response. In addition, corresponding bone formation was measured for **(E)** HO and **(F)** fracture using microCT. Bone volume is depicted in the graphs. Significant differences (p<0.5) were calculated using an ANOVA with Tukey correction. Symbols represent sample data points, and horizontal lines depict the mean. Standard error of the mean (SEM) is shown by the capped vertical line. *p < 0.05, **p < 0.01.

ADRβ3+ macrophages express several transcripts associated with M2 macrophages including Mrc1, Arg1, Tlr2, Cd83, and Ucp2 (**Figure 6A**). Since activation of the M1 proinflammatory phenotype has been shown to suppress M2 macrophages, we next attempted to polarize macrophages in the mouse to an M1 phenotype, to suppress the formation of M2 regenerative macrophages. LPS was delivered to mice to activate a proinflammatory phenotype. As predicted, ADRβ3+ macrophages showed a significant decrease in number when treated with LPS as compared with the same population isolated from saline-treated animals (**Figure 6C**). This suggests that ADRβ3+ macrophages either polarize towards the M1 phenotype potentially altering their phenotype and down-regulating ADRβ3 or they fail to expand or recruit to the site of HO. The resultant bone formation was also significantly suppressed in the LPS-treated as compared to saline-treated mice (**Figure 6E**), suggesting that M2 macrophages may play a key functional role in this process. The ADRβ3+ macrophages present in the fracture callus (see below) also express several M2 macrophage markers, sometimes only in the macrophage cluster (FC5), but sometimes also in the other blood lineages (data not shown) present (**Figure 6B**). When mice with a fracture were treated with LPS, the ADRβ3+ macrophages in callus also showed a significant decrease in number when treated with LPS as compared with the same population isolated from saline-treated animals (**Figure 6D**), which was again reflected in a significant decrease in bone volume (**Figure 6F**).

### The Amount of Heterotopic Bone Increases Upon Specific Destruction of ADRβ3+ Macrophages

**Figure 7:**
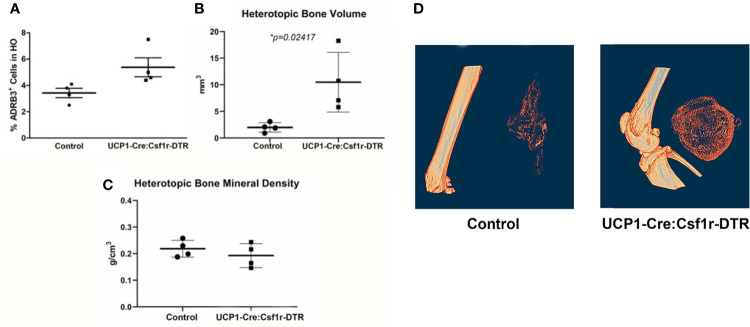
The amount of heterotopic bone increases upon specific destruction of ADRβ3+ macrophages. Ucp1-Cre : Csfr1 ^floxSTOPflox^DTRmcherry mice were injected with Dta and with AdBmp2-transduced cells on day 0. On day 4 the mice were euthanized and the amount of Ucp1+ cells in heterotopic bone was determined as well as the bone volume and bone mineral density. Panel **(A)** shows the percentage of Adrb3+ cells present in the control compared to the percentage of Adrb3+ cells present in Ucp1-Cre:Csf1r-DTR, **(B)** shows the heterotopic bone volume in mm3 mice present in the Control as compared to that present in the Ucp1-Cre:Csf1r-DTR mice, and **(C)** shows the heterotopic bone mineral density in Control mice versus Ucp1-Cre:Csf1r-DTR. **(D)** shows the actual slices of the amount of bone in mice injected with Bmp2 *versus* Dta-treated mice injected with Bmp2.

Since macrophages readily alter their phenotype in response to the microenvironment, we attempted to ablate these specific cells, to identify any downstream effects that may alter HO. To accomplish this, we utilized a two-gene system in a diphtheria toxin receptor-mCherry fusion protein (DTR-mCherry) preceded by a loxP-flanked in which Cre recombinase is expressed through a UcP1 promoter (UcP1-Cre) and was crossed to a mouse possessing transcriptional stop element under the control of the Csf1r promoter. HO was induced in these mice by injection of AdBMP2-transduced cells and then either diphtheria toxin or saline (control). Approximately 4 days later, cells associated with HO were isolated and subjected to flow cytometry to detect ADR*β*3+ cells. ADRβ3+ cells showed no change compared to the control mice (**Figure 7A**), even though these mice were euthanized 4 days after addition of BMP2, which is close to the peak of ADR*β*3+ cells (**Supplemental Figure 1A**). However, there was a significant increase in bone volume in the group where UcP1+ Csfr1+ macrophages were selectively depleted (**Figure 7B**). Bone density however was unchanged between groups (**Figure 7C**). Alternatively, DTR is a fusion protein with mcherry, and these cells were absent in the diphtheria toxin A treated samples but present in the control, which was saline treated (see Materials and Methods). Thus, cells were depleted as expected, but this depletion resulted in no change in the ADRβ3+ cells with the amount of heterotopic bone formed significantly increased (**Figure 7B**). **Figure 7D** shows a single section through both the control and the Ucp1-Cre mouse. The data collectively are consistent with our former hypothesis (6) that compromise of the ADRβ3+ cells results in a compensating oxidation and beiging of white fat. Although this makes more hypoxic areas resulting in more bone formation, it does so at the expense of the ability to control reactive oxygen species through uncoupling proteins (41), and most importantly, since skeletal bone in a Ucp1 compromised *(Misty*) mouse was extremely osteoporotic (42), perhaps the quality of the bone that is made in the absence of Ucp1 is abnormal even though there is more of it.

### ADRβ3+ Macrophages Are Also Present in Fracture Callus

To determine if these macrophages are associated with other types of bone formation, mice received a femur defect, and cells were then isolated from the resultant fracture callus. Like HO, ADRβ3+ cells were identified in callus and found to be transient with their peak expression approximately 4 days after introduction of the defect (**Supplemental Figure 2B**).

**Figure 8:**
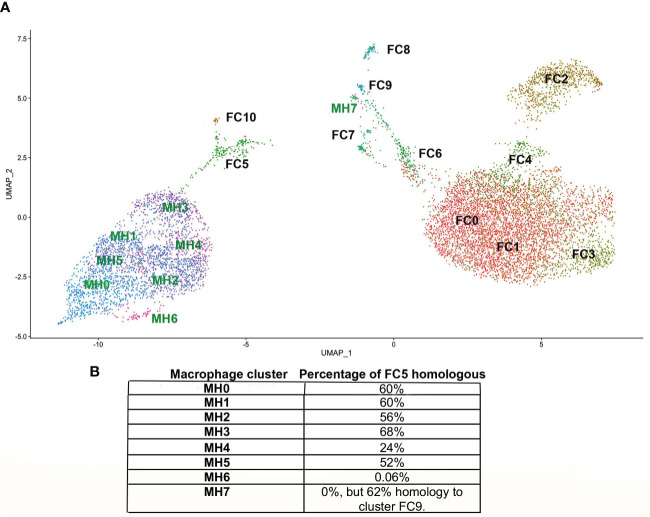
**(A)** Co-embedding of the Seurat from HO with that from fracture callus. In order to more accurately assess common features between the MH and FC clusters they were co-embedded by first processing the gene activity matrix in order to find anchors between cells in the MH and FC datasets. These anchors were then used to transfer the data by the standard workflow in Seurat to plot the co-embedded dataset. **(B)** The percentage of the top 50 transcripts of each MH cluster present in FC5 are indicated. Although cluster MH7 has no transcripts in FC5, it has 62% of its transcripts present in FC9.

The ADRβ3+ cells were then isolated by FACS (**Supplemental Figure 2D**) and their RNA sequenced using scRNAseq. The data was analyzed using the Seurat (v3) algorithm, which clustered the resultant cells into 11 groups (**Figure 1B**). Only one cluster (FC5) expressed transcripts associated with macrophages (**Figure 2D**) and osteoclasts (**Figure 2F**). In order to observe the similarities and differences between macrophage transcripts from fracture callus and those from HO, the Seurat data from each were co-embedded (15) (**Figure 8A**). Co-embedding, which is a part of Seurat v3, forms transfer anchors between two datasets and harmonizes the data such that the two can be directly compared (15). The result shows that cluster FC5 is very similar to clusters MH0-MH5 because FC5 migrates close to these clusters when co-embedded. Surprisingly, cluster MH7 is now very close to FC9, which both have markers of hemogenic endothelium, suggesting perhaps there is a relationship between these macrophages rather than their presence as simple contaminants (**Figure 8A**). When the transcripts comprising cluster FC5 were directly compared to those of clusters MH0-MH7 (**Supplemental Table 2**), although a great deal of similarity was apparent, transcripts corresponding to cluster MH6 and MH7 were totally absent from FC5, and only 24% of the transcripts from cluster MH4 corresponded to cluster FC5. Although transcripts from MH7 were totally absent from FC5, 62% of the MH7 transcripts were present in cluster FC9. This again shows the similarity between cluster MH7 and FC9, both having markers of hemogenic endothelium. The rest of the MH clusters had between 52-68% of their transcripts present in FC5 (**Figure 8B**). The absence of a highly replicating cell, like MH6, and the absence of some of the more highly expressed transcripts in the MH clusters in FC5 may indicate that the fracture repair is at a later stage of bone formation than HO.

### Pseudotime Analysis of the Data Using Monocle 3

Pseudotiming of the data was performed using the Monocle (version 3) algorithm (43). To convert Monocle, to a Seurat (version3) object, it was first converted to a Monocle Cell Data Set. We plotted a trajectory for each of the clusters associated with HO using Monocle 3. These trajectories depict the ordering of the clusters in pseudotime (**Supplemental Figure 4**). The trajectory determination orders the clusters from earliest to latest.

All clusters for MH appear to be connected (**Supplemental Figure 4B**). The earliest cell type in the genesis of ADRβ3+ macrophages is MH6, which appears to undergo further changes to form other M2 macrophages (**Supplemental Figure 4C**). It is interesting that MH6 shows high homology to the Replicating Stem-like Cell, which is a stem-like cell for osteogenesis that goes next to the Chondro-Osseous Progenitor (COP) through an epithelial to mesenchymal transition (44). The MH6 progenitor leads directly to MH7 (**Supplemental Figure 4**), that contains markers of hemogenic endothelial cells and MH7 is the progenitor for MH0. Cluster FC9 also contains markers of hemogenic endothelial cells (**Supplemental Table 1**) and when the Seurat plots of HO and fracture callus are co-embedded (**Figure 8A**), cluster MH7 and FC9 lay almost directly on top of each other, indicating their similarity.

## Discussion

Previous studies in our laboratory had identified transient brown adipocyte-like cells that appear during HO in both humans (7) and mice (6, 8). Our data suggested that they metabolically regulate oxygen tension and angiogenesis supporting a key role in patterning the newly forming tissues (6–8). Here, we extend this knowledge to show that these cells are macrophages that recruit to the site of new bone formation. These ADRβ3+ macrophages are also transient, appearing within 48 hours of induction of bone formation and disappearing as quickly as six days later. While the transcriptome supports their expression of angiogenic factors, M2 macrophages have previously been found to regulate angiogenesis during tissue regeneration (45, 46) and vessel remodeling (47). Analysis of the phenotypic markers suggests that these cells are more polarized towards the M2 phenotype. M2 macrophages have also been shown to possess elevated mitochondria and express Ucp2. While Ucp1 expression has only been reported in brown adipocytes, here we extend this finding to show that M2 macrophages can also transiently express this uncoupling protein, suggesting a possible functional role for uncoupled metabolism. While emphasis has been placed on reducing reactive oxygen production, uncoupled respiration can also reduce the availability of local oxygen to other cells (6), thereby altering their metabolism. Since recent studies in cellular reprogramming suggest a key role for glycolysis in this process (48–50), perhaps another role for these cells is in turning on this switch through depletion of local oxygen.

These ADRβ3+ macrophages also express several adipogenic factors, including the adiponectin receptor, suggesting that these cells can be regulated by adipokines. In our previous experiments, we utilized a mouse (Dock 7 deficient) that lacked brown adipocytes and found that these transient brown adipocyte-like cells or macrophages were not able to express Ucp1, and surprisingly, more heterotopic bone formed in these mice. Analysis of the tissues suggested that adjacent white adipocytes had compensated and had undergone oxidation in a fat burning process termed “beiging” (51). To confirm that this finding applies to depletion of these ADRβ3+ macrophages, HO was established in a unique mouse model which would selectively ablate cells that were Ucp1^+^ Csf1r^+^ by driving them to express the diphtheria toxin receptor. Since mice do not express this receptor normally, only Ucp1+ Csf1r+ cells, which now express the receptor, will be ablated by delivery of diphtheria toxin A. Surprisingly, in these studies, mice had significantly more heterotopic bone than their counterparts that received saline. The data suggests that by lowering oxygen in the tissues they may drive early angiogenesis, and their transient nature may enable them to easily terminate this process. However, it appears that depletion of all macrophages with clodronate showed a significant reduction in HO, while depletion of only the M2 population seems to have the opposite effect increasing the amount of bone formed. Although this suggest that the M2 macrophages are unique and different from the total population, there is no definitive proof that the ADRβ3+ population is essential. More studies are warranted to confirm their mechanism of action. Alternatively, considering our current finding that these cells are M2 macrophages, suggests that M2 macrophages and adipocytes have significant cross talk, perhaps to regulate metabolism during tissue regeneration.

Another surprising finding is the similarity of ADRβ3+ macrophages to CNS- and PNS-associated macrophages. The CNS macrophages (Bams) are associated with boundary regions of the highly protected brain tissue and are thought to play a role in maintaining the protective barrier (52). Surprisingly, ADRβ3 is a hallmark of brown adipocytes, which are believed to be generated by activation of the sympathetic nervous system. Our own studies linked elevated circulating noradrenaline with the appearance of these cells (8) and blocking substance P effectively stops their formation (53, 54). Perhaps these M2 macrophages are a tissue resident population that responds to neuroinflammatory signaling to support tissue regeneration. Further, during these studies, we showed that ADRβ3+ cells were associated with peripheral nerves (8), suggesting that these cells could be recruited from peripheral nerve macrophages, which possess a similar transcriptome.

These cells not only have a unique metabolism, but also possess the key hallmarks of macrophages. They are able to undergo M1-M2 polarization and show toxicity to clodronate liposomes indicating that they are phagocytes. Further, the transient nature of these cells is most likely due to the cessation of ADRβ3 and Ucp1 expression rather than the disappearance of the cells themselves. Adrenergic receptors are known to undergo β-arrestin-mediated desensitization by endocytosis of the receptor (55). Perhaps these cells undergo further differentiation into other types of macrophages. One potential population could be osteoclasts, since several osteoclast-specific transcripts were observed in some of the later clusters associated with HO.

While these cells appeared to possess many macrophage properties, when they are compared with BMDMs and microglia, there are several differences. Beyond the differences in surface marker expression, one additional difference is their viability in culture. Both primary BMDMs and microglia upon isolation were able to be cultured through inclusion of colony stimulatory factor in the medium. The addition of this factor did not influence viability of the ADRβ3+ cells, showing they are distinct from these other populations.

Since the macrophages appeared to be M2-like, we next tested whether delivery of LPS to either the site of *de novo* bone formation or fracture callus could suppress their phenotype leading to downstream effects on bone formation. As predicted, the number of ADRβ3^+^ macrophages was significantly reduced, with a correlating reduction in overall bone formation in both processes. Thus, enforcing the M1 phenotype appears to suppress these macrophages and results in reduced bone formation. While we have not been able to selectively remove only this population of macrophages, the data collectively suggests that they may play a key role in regulating bone formation.

Surprisingly, these ADRβ3+ M2-like macrophages were also observed in a bone defect repair model. There was significant overlap in their transcriptomes suggesting they are similar, but this may be a transient phenotype as observed in the HO model. An intriguing question is the importance of these macrophages to skeletal bone repair. They express Lrp1 in almost every cluster during HO, which was previously shown by Vi et al. (56) to be an essential protein in macrophages that assist in fracture repair. The data collectively suggests the presence of a unique lipogenic macrophage that may be capable of unique metabolic regulation that may support two different types of bone formation. Since macrophages are known for patterning their environment, perhaps these cells are dissimilar, but reacting to similar metabolic cues to assist in bone formation. In conclusion, these ADRβ3^+^ lipogenic macrophages appear to be similar to M2 macrophages and an essential accessory cell for bone formation.

Recent studies in both HO and fracture repair have also shown the contribution of macrophages to these processes. Sorkin et al. (57) recently reported specific macrophage populations that they proposed drove heterotopic ossification and there are some similarities to the studies reported here. However, there are major differences including the low level of expression of F4/80 and Cd11b observed on the Adrb3+ macrophages compared to the population described by Sorkin et al. (57) that clearly retain high-level expression of F4/80nd Cd11b. In the studies presented here, we did not look at all macrophages but rather attempted to limit our analysis to the brown adipocyte-like cells we had previously analyzed. However, we can confirm that there were other macrophage populations present that were Cd11b positive, and they may be more similar to the macrophages described by Sorkin et al. (57).

Vi et al. (56) also demonstrated the essential nature of macrophages in fracture repair. Like the studies of Sorkin et al. (57), experiments using clodronate liposomes to deplete all macrophages resulted in suppression of transcripts were highly expressed in all but cluster MH7 in the macrophages associated with HO as well as cluster 5 macrophages (FC5) associated with fracture callus. These findings suggest that the Lrp1- expressing macrophages play a key lipid regulatory role. Transcripts like Cav1 (MH7) a master regulator of caveolae (58) and Cav2 (MH0, 6, and 7), are found mostly in the earlier clusters. Cav1 is involved in the regulation of lipolysis through perilipin and hormone sensitive lipase. Perilipin and Plin2 are also present in the fracture callus macrophage cluster MC5. Finally, the fracture callus also contains Abhd5 (MC10), which is an acyltransferase for the synthesis of phosphatidic acid, the major intermediate in membrane and storage lipid biosynthesis and it functions as a co-activator of adipocyte triglyceride lipase (Pnpla2, MC9). Neither Abhd5 nor Pnpla2 are present in macrophages appearing during *de novo* bone formation, which may be in part due to the time of the cell isolation. Thus, macrophage plasticity may play a key role in assisting fracture repair during this process through regulation of lipid.

## Data Availability Statement

The original contributions presented in the study are publicly available. This data can be found here: https://www.ncbi.nlm.nih.gov/geo/query/acc.cgi?acc=GSE185500.

## Ethics Statement

The animal study was reviewed and approved by Institutional Animal Care and Use Committee (IACUC), Baylor College of Medicine.

## Author Contributions

EO-D: conception and design, collection and/or assembly of data, and manuscript writing. JM collection and/or assembly of data. ES: collection and/or assembly of data, data analysis and interpretation, manuscript writing, and conception and design. ZG: collection and/or assembly of data, data analysis and interpretation, and manuscript writing AD: bioinformatics data analysis, conception and design, manuscript writing, financial support, and final approval of manuscript. All authors contributed to the article and approved the submitted version.

## Funding

This work was supported by grants from the National Institute of Arthritis and Musculoskeletal and Skin Disease (R01AR066556) and the U.S. Department of Defense (W81XWH-16-1-0649) and (W81XWH-17-01-0628).

## Conflict of Interest

The authors declare that the research was conducted in the absence of any commercial or financial relationships that could be construed as a potential conflict of interest.

## Publisher’s Note

All claims expressed in this article are solely those of the authors and do not necessarily represent those of their affiliated organizations, or those of the publisher, the editors and the reviewers. Any product that may be evaluated in this article, or claim that may be made by its manufacturer, is not guaranteed or endorsed by the publisher.

## Supplementary Material

Supplementary Figure 1 Immunohistochemisty for Cd14 and Adrb3 in mouse lungs. Mouse lungs were infected with 5 x 10^12^ AdBmp2. After 4 days the mice were euthanized. The lungs were fixed in formalin and then embedded in paraffin. Sections were stained with both anti Cd14 (green) and anti Adrb3 (red).

Supplementary Figure 2 Transient nature of ADRβ3+ cells during HO. **(A)** Quantitation of ADRβ3+ cells using flow cytometry at different time points during HO. **(B)** Quantitation of ADRβ3+ cells using flow cytometry at different time points during fracture repair. Representative flow cytometry gating strategies for the isolation of the ADRβ3+ cells from **(C)** HO and **(D)** fracture callus for use in scRNAseq.

Supplementary Figure 3 The transcriptome of MH6 has a high content of transcripts involved in growth and replication. The transcriptome of MH6 was uploaded into Pathway Studio (Elsevier, Amsterdam) for analysis.

Supplementary Figure 4 Pseudotiming of the clusters obtained from ADRβ3+ cells isolated from HO using the Monocle v.3 algorithm. **(A)** Each of the ADRβ3+ cell clusters associated with HO was assigned a specific marker using the top_specific_marker_IDs algorithm in Monocle 3. **(B)** A trajectory of cell types was created using Monocle 3. **(C)** A dichotomy tree is indicated for HO.

Supplementary Figure 5
**(A)** ADRβ3+ macrophages possess a similar transcriptome to boundary-associated macrophages. Table depicts highly expressed transcripts in common between ADRβ3+ cells isolated from HO and BAM cells. The number depicts the position of the transcripts with the most highly expressed being represented as (1). **(B)** ADRβ3+ macrophages induced during heterotopic ossification express all of the top 30 transcripts of PNS macrophages. a. The top 30 PNS macrophage markers as determined by Wang et al. (25) were used to search the transcripts found in MH0 to MH7. Each marker was found in all or almost all cell types MH0 to MH7. b. The top 30 PNS macrophage markers as determined by Wang et al. were used to search the transcripts found in FC0 to FC10.

Supplementary Table 1 Top 50 transcripts in each cluster MH0 to MH7 and FC0 to FC10. The top 50 transcripts are listed for each cluster.

Supplementary Table 2 Macrophage markers (MH0-MH6) in FC5. The transcripts from FC5 were compared to the top 50 transcripts of each cluster MH0 to MH7. A plus sign indicates the presence of a corresponding transcript in FC5, while a minus sign indicates the absence of a corresponding FC5 transcript.

## Abbreviations

Bmp2, Bone morphogenetic protein type 2; scRNAseq, Single-cell RNA sequencing; LPS, Lipopolysacheride; HO, Heterotopic ossification; BAT, like cell-Brown-adipocyte-like cell; BAMs, Brain-associated macrophages; Csf1, Colony stimulating factor 1; Gfp, green fluorescent protein; HBSS, Hank’s buffered saline solution; PCR, polymerase chain reaction; AdBmp2, An adenovirus vector engineered to enable production of Bmp2 upon transduction of a cell substrate; Hif1, hypoxia inducible factor 1; FACS, fluorescent-activated cell sorting; IACUC, Institutional Animal Care and Use Committee; CNS, central nervous system; PNS, Peripheral-nervous system.
