# Supplementary figures and images for: A Population of M2 Macrophages Associated With Bone Formation
Source: Front Immunol. 2021 Oct 12;12:686769. doi: 10.3389/fimmu.2021.686769 (PMC8547272; doi:10.3389/fimmu.2021.686769)

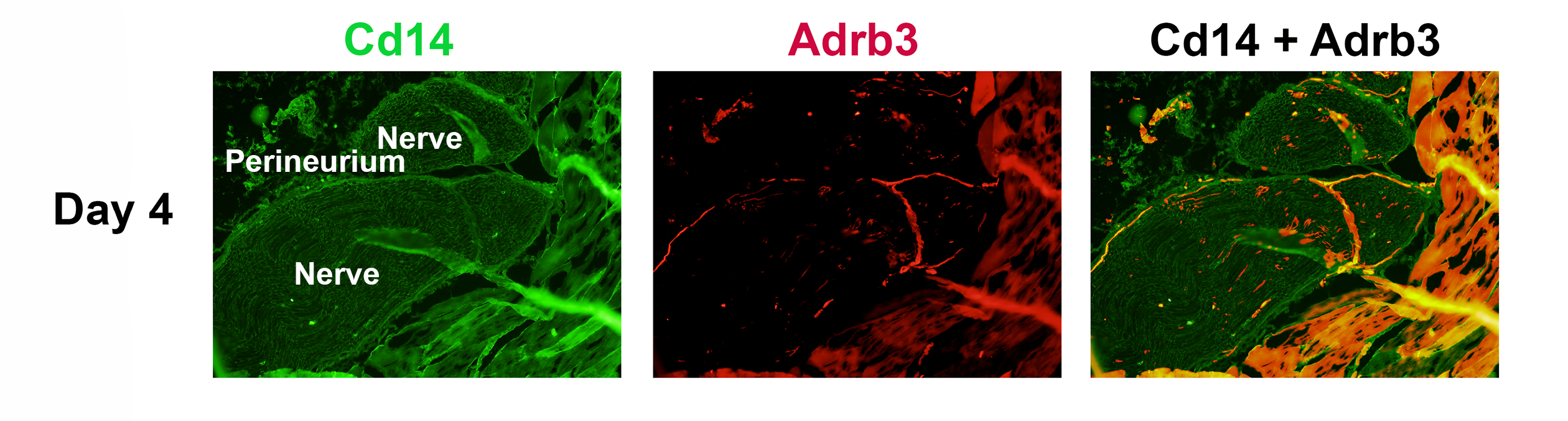

Supplement: Supplementary Figure 1 — Immunohistochemisty for Cd14 and Adrb3 in mouse lungs. Mouse lungs were infected with 5 x 10^12^ AdBmp2. After 4 days the mice were euthanized. The lungs were fixed in formalin and then embedded in paraffin. Sections were stained with both anti Cd14 (green) and anti Adrb3 (red). [file Image_1.png]

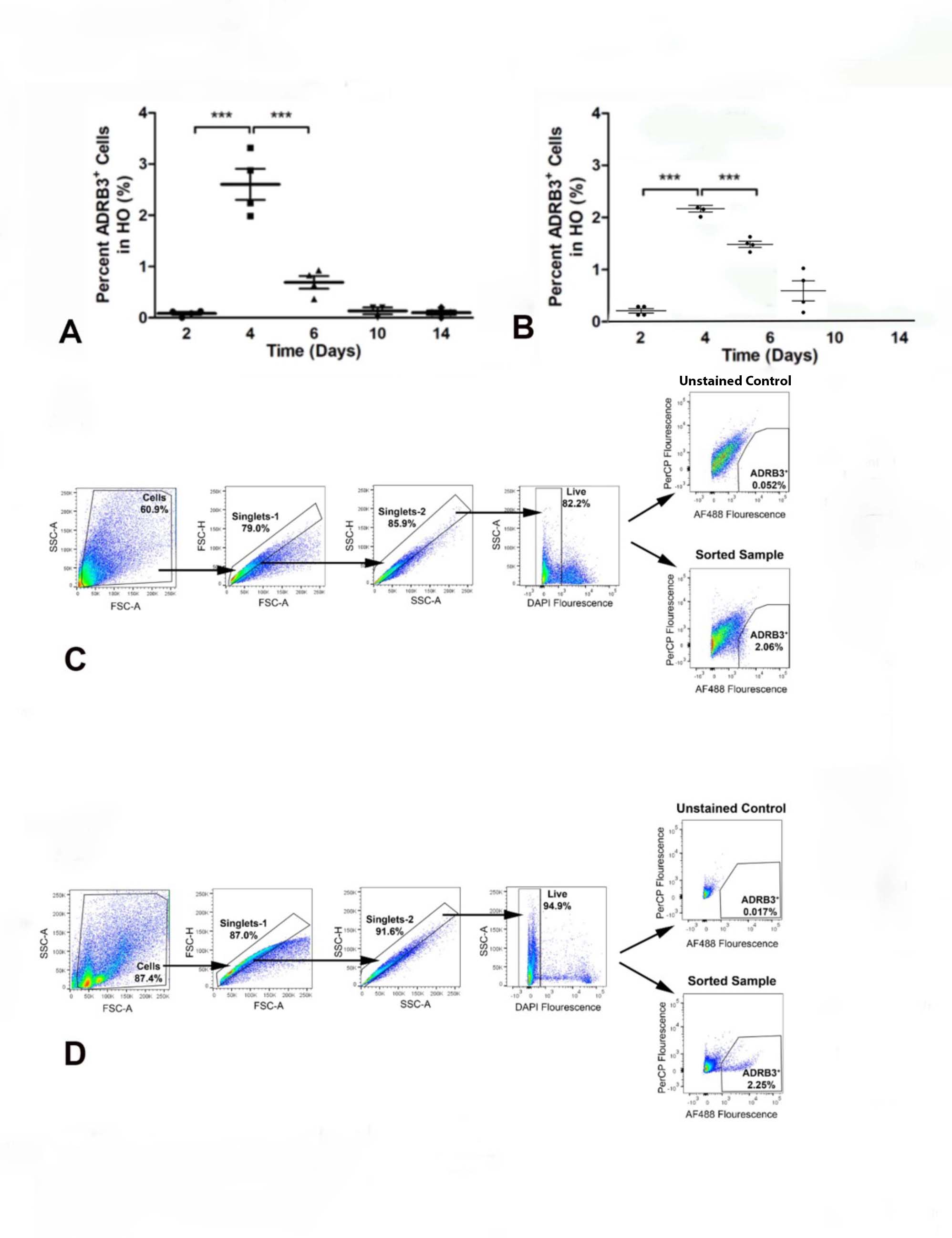

Supplement: Supplementary Figure 2 — Transient nature of ADRβ3+ cells during HO. (A) Quantitation of ADRβ3+ cells using flow cytometry at different time points during HO. (B) Quantitation of ADRβ3+ cells using flow cytometry at different time points during fracture repair. Representative flow cytometry gating strategies for the isolation of the ADRβ3+ cells from (C) HO and (D) fracture callus for use in scRNAseq. [file Image_2.jpeg]

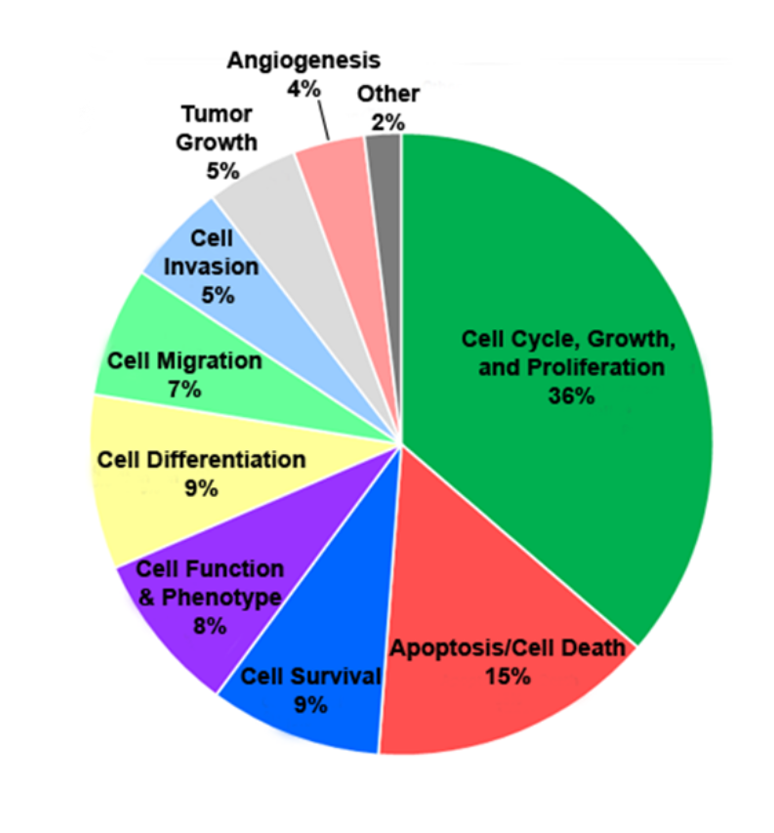

Supplement: Supplementary Figure 3 — The transcriptome of MH6 has a high content of transcripts involved in growth and replication. The transcriptome of MH6 was uploaded into Pathway Studio (Elsevier, Amsterdam) for analysis. [file Image_3.tif]

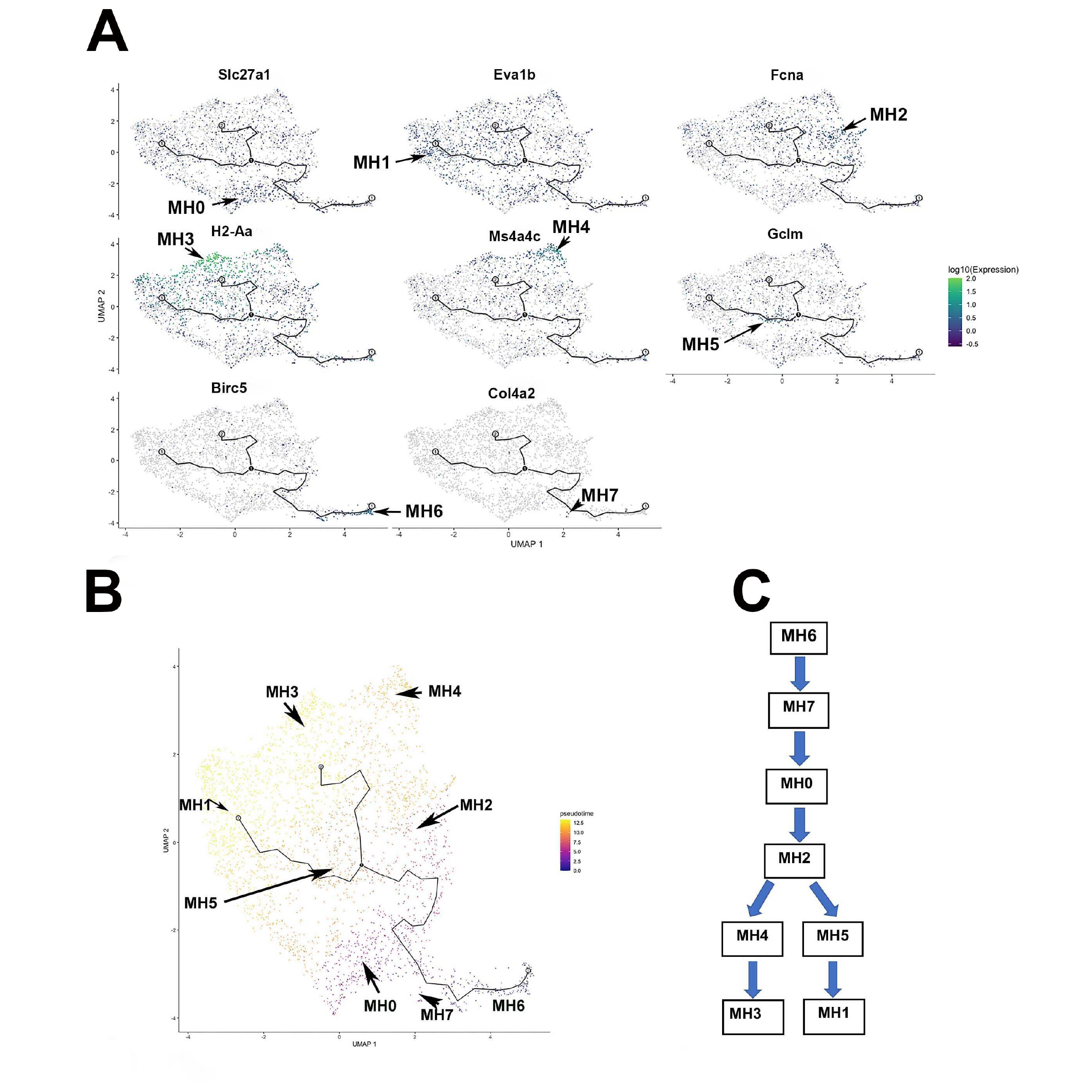

Supplement: Supplementary Figure 4 — Pseudotiming of the clusters obtained from ADRβ3+ cells isolated from HO using the Monocle v.3 algorithm. (A) Each of the ADRβ3+ cell clusters associated with HO was assigned a specific marker using the top_specific_marker_IDs algorithm in Monocle 3. (B) A trajectory of cell types was created using Monocle 3. (C) A dichotomy tree is indicated for HO. [file Image_4.tif]

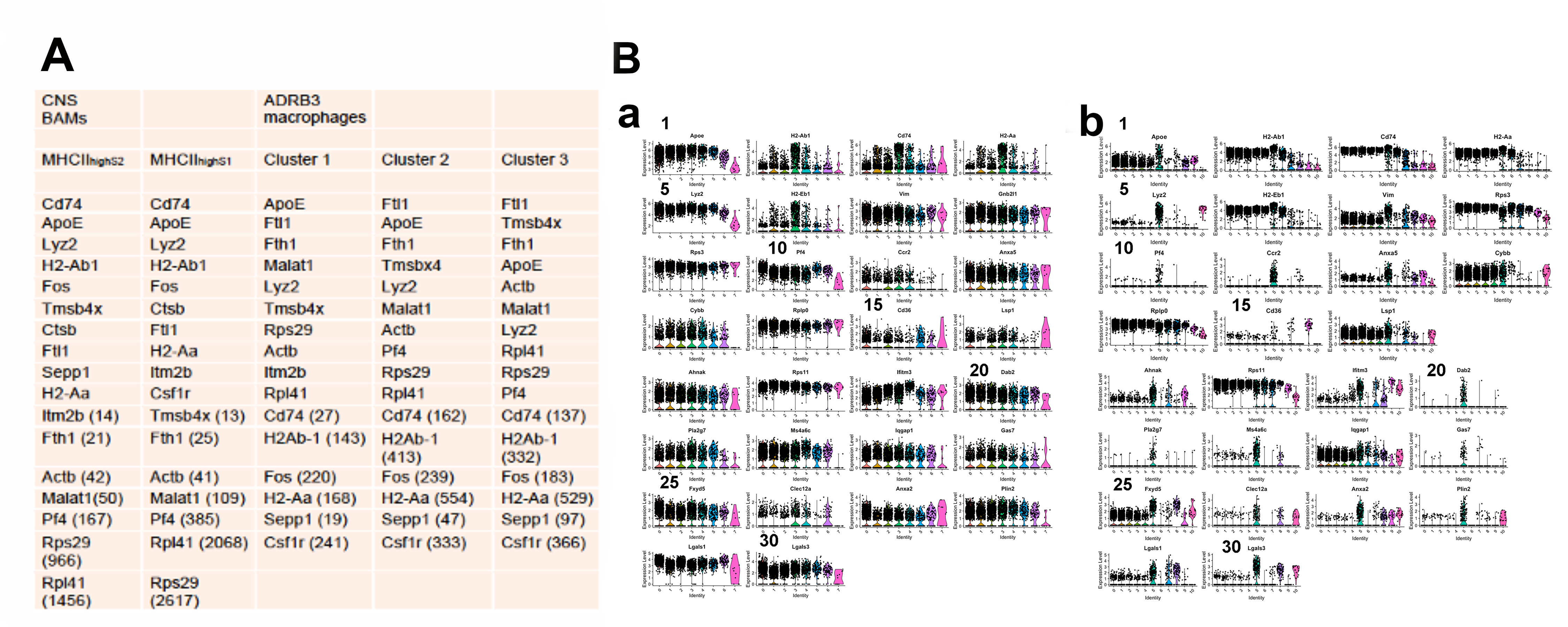

Supplement: Supplementary Figure 5 — (A) ADRβ3+ macrophages possess a similar transcriptome to boundary-associated macrophages. Table depicts highly expressed transcripts in common between ADRβ3+ cells isolated from HO and BAM cells. The number depicts the position of the transcripts with the most highly expressed being represented as (1). (B) ADRβ3+ macrophages induced during heterotopic ossification express all of the top 30 transcripts of PNS macrophages. a. The top 30 PNS macrophage markers as determined by Wang et al. (25) were used to search the transcripts found in MH0 to MH7. Each marker was found in all or almost all cell types MH0 to MH7. b. The top 30 PNS macrophage markers as determined by Wang et al. were used to search the transcripts found in FC0 to FC10. [file Image_5.tif]
