# Supplementary material for: A Population of M2 Macrophages Associated With Bone Formation
Source: Front Immunol. 2021 Oct 12;12:686769. doi: 10.3389/fimmu.2021.686769 (PMC8547272; doi:10.3389/fimmu.2021.686769)
Supplement: Supplementary Table 1 — Top 50 transcripts in each cluster MH0 to MH7 and FC0 to FC10. The top 50 transcripts are listed for each cluster. [file Table_1.docx]

Supplemental Table 1

| **MH0** | **MH1** | **MH2** | **MH3** | **MH4** |
| --- | --- | --- | --- | --- |
|  |  |  |  |  |
| Sepp1 | Rps29 | Folr2 | H2-Aa | Ifit3 |
| Mif1 | Rps27 | Wfdc17 | H2-Eb1 | Ifit2 |
| Pf4 | Rpl37a | Sepp1 | Cd74 | Isg15 |
| Gapdh | Rpl35a | Ccl6 | H2-Ab1 | Ms4a4c |
| Pdpn | Rps14 | Fcgrt | Tmem176b | Irf7 |
| Pkm | Rpl13a | Ednrb | Tmem176a | Rsad2 |
| Lgals1 | Capg | Ltc4s | H2-DMb1 | Zbp1 |
| Aldoa | Lgals3 | Fcna | H2-DMa | Pydc4 |
| S100a4 | Lgals1 | Thb1 | Ctsh | Ifit1 |
| Arg1 | Anxa2 | Cbr2 | Lgals1 | Ifitm3 |
| S100a6 | Ppia | Clec10a | H2-D1 | Rtp4 |
| Tpi1 | S100a4 | Tslp | Apoe | Gm4955 |
| Ifitm3 | Prdx1 | Timp2 | Plbd1 | Phf11b |
| Pltp | S100a6 | Atf3 | Trf | Phf11d |
| Eno1 | Rps9 | Lyve1 | Ubb | Oas3 |
| Ly6e | Calm1 | Rcn3 | Cd83 | Ly6a |
| Bnip3 | Pfn1 | Gas6 | Pla2g7 | Ube2l6 |
| Pgam1 | B2m | Igf1 | Il1b | Pyhin1 |
| Vegfa | Cfl1 | Cxcl16 | Arl5c | Xaf1 |
| Capg | Ctsl | Pltp | Ifitm2 | Mnda |
| Jund | S100a10 | Ifitm3 | Srgn | Ly6e |
| Ldha | S100a11 | Serpinb6a | Lyz2 | Bst2 |
| Anxa2 | Ldha | Id2 | Cst3 | Plac8 |
| Fn1 | H2-D1 | F13a1 | Spp1 | Oasl1 |
| Clec4d | Clic1 | Pepd | Syngr1 | Irgm1 |
| Basp1 | Gapdh | Aldh2 | Cyp4f18 | Ifi204 |
| Tmem176b | Eno1 | Ccl8 | Cd52 | Fcgr1 |
| Serinc3 | Cd68 | Clec4d | Mif | Mndal |
| Itm2b | Ms4a6d | Fth1 | Pf4 | Parp14 |
| Wfdc17 | Ctss | Ap2m1 | Pltp | Ifi47 |
| Clec10a | Atp5b | Mt1 | Hspa8 | Tor3a |
| Lat2 | Pkm | Ccl2 | Pdpn | Ifi203 |
| Slc16a3 | Tmsb10 | Selenbp1 | Ndufa2 | Sp110 |
| Adam8 | Rps12 | Atox1 | Atp5e | Trim30a |
| Ccl6 | Rps21 | Cd52 | Unc93b1 | Ifi27l2a |
| Trf | Jund | Basp1 | Uqcr11 | Oas1a |
| Mxi1 | Myl12a | Btg1 | Ly86 | Fcgr4 |
| Tmem176a | Ubb | Vegfa | Serpinb6a | Ifi35 |
| Pfn1 | Uqcrb | Fxyd2 | Mrc1 | Stat1 |
| Id2 | Tpi1 | Ly6e | Cela1 | Slfn4 |
| Ctla2a | Myl6 | Snx5 | Cd14 | Psmb8 |
| Fxyd5 | Pgam1 | Ccl9 | F13a1 | Tap1 |
| Aprt | Tspo | Pmp22 | Timp2 | H2-T22 |
| Igf1 | Tagln2 | Zfand5 | Snx3 | Ifih1 |
| Cd74 | Arpc3 | Klf2 | Ctsc | B2m |
| Thbs1 | Emp3 | Gda | Gaddd45b | Sp100 |
| S100a11 | Fxyd5 | Hint1 | Ifrd1 | Rnf213 |
| Pgk1 | Cotl1 | Cd302 | Cxcl16 | Lgals3bp |
| Unc93b1 | Cbr2 | Pfkfb3 | Gapdh | Pnp |
| S100a10 | Psma2 | Npl | Rnase4 | Dck |

| **MH5** | **MH6** | **MH7** |
| --- | --- | --- |
| Slc40a1 | 2810417H13Rik | Col15a1 |
| Gclm | Nusap1 | Tmem47 |
| Ftl1 | Birc5 | Clec14a |
| Slc48a1 | Pbk | Abi3bp |
| Fth1 | Top2a | Erg |
| Hmox1 | Cdca3 | Cyr61 |
| Gm10116 | Ccna2 | Plpp3 |
| Prdx1 | Lockd | Hspg2 |
| Creg1 | Cdkn3 | Nfib |
| Gsr | Prc1 | Cd200 |
| Srxn1 | Tk1 | Wwtr1 |
| Clec4n | Ube2c | Timp3 |
| Lipa | Fam64a | Sparcl1 |
| Slc7a11 | Racgap1 | Ablim3 |
| Slc11a1 | Ccnb2 | Nid1 |
| Pgd | Rrm2 | Mecom |
| Blvb | Spc24 | Trp53i11 |
| Htatip2 | Cdk1 | Col4a2 |
| Clec4d | Cdca8 | Mmrn2 |
| Mgst1 | Ccnb1 | Tm4sf1 |
| Cyb5a | Stmn1 | Spint2 |
| Gstm1 | Cks1b | Ramp2 |
| Ctsd | Hist1h2ap | Npdc1 |
| Txnrd1 | Tpx2 | Tinagl1 |
| Cd36 | Cenpf | Sdc2 |
| Cat | Cenpm | Fbln2 |
| Gclc | Uhrf1 | Lpar4 |
| Txn1 | Mad2l1 | Emcn |
| Ednrb | Cenpw | Sdpr |
| Ninj1 | Knstrn | Fxyd6 |
| Sqstm1 | Smc2 | Lims2 |
| Akr1b8 | Tyms | Gpihbp1 |
| Pf4 | Mki67 | Fscn1 |
| Taldo1 | Tmpo | Cnn3 |
| Igf1 | Spc25 | Igfbp 5 |
| G6pdx | Asf1b | Apbb2 |
| Pla2g7 | Mcm5 | Cldn5 |
| Msrb1 | H2afx | Fermt2 |
| Abcc1 | Ezh2 | Cd34 |
| Tnfaip2 | Lsm2 | Jam2 |
| Gdf15 | Smc4 | Tmem158 |
| Aldoa | Gmnn | Nedd4 |
| Por | Tacc3 | Emp2 |
| Akr1a1 | Tuba1b | Igfbp7 |
| Pdlim4 | Cks2 | Cdh5 |
| Gde1 | Ptma | Bcam |
| Adam8 | Hmgn2 | Ptrf |
| Prdx6 | H2afz | Kdr |
| S100a4 | Nucks1 | Ly6c1 |
| Cstb | Hmgb1 | Cttn |
| Cela1 | Gm10282 | Fxyd1 |

| **FC0** | **FC1** | **FC2** | **FC3** | **FC4** |
| --- | --- | --- | --- | --- |
|  |  |  |  |  |
| Rps29 | Eif1 | Hspa1a | Myc | Ifit3 |
| Rps24 | Sub1 | Hspa1b | Bcl2a1b | Ifi47 |
| Rpl8 | Vps37b | Gm26825 | Egr3 | Zbp1 |
| Rpl35a | Cd83 | Iglc3 | Nfkbid | Tor3a |
| Rps20 | Junb | Iglc2 | Cd83 | Rtp4 |
| Rps15a | H3f3b | Ltb | Bcl2a1d | Irgm1 |
| Rpl27a | Rps2 | Jun | Rel | Slfn5 |
| Rpl9 | Ncl | Pxdc1 | Irf4 | Parp14 |
| Rps27 | Ezr | Arpc5l | Nr4a3 | Phf11b |
| Rpl13 | Ubc | Cd79 | Pim | Isg15 |
| Rpl37a | Rel | Swap70b | Nr4a1 | Ifi206 |
| Rpl21 | Rpl12 | Ms4a1 | Gadd45b | Irf7 |
| Rps3a1 | Ccr7 | Bcl11a | Nfkbia | Ifi214 |
| Rps7 | Satb1 | Iglc1 | Rilpl2 | Ifi203 |
| Gdi2 | Cnbp | Ly6d | Ifrd1 | Xaf1 |
| Rpl30 | Ptma | Pfn1 | Gpr183 | Ifi27l2a |
| Rpl37 | Jund | Ptpn 6 | Srgn | Isg20 |
| Rps4x | Dennd4a | Ighm | Kdm6b | Ifi213 |
| Rps21 | Rpl22l1 | Coro1a | Traf1 | Slfn2 |
| Rpsa | Dad1 | Ifi203 | Gnl3 | Oasl1 |
| Rps19 | Tubb4b | Cd52 | Ankrd33b | Trim30a |
| Fau | Tuba4a | Tnfrsf13b | Marcksl1 | Shisa5 |
| Rpl19 | Nr4a1 | Sh3bp5 | Hspa5 | Stat1 |
| Rpl18a | Cytip | Cd37 | Ncl | Ms4a4c |
| Rps5 | Pnrc1 | Rac2 | Rasgef1b | Ly6a |
| Rps8 | Srsf5 | Klf2 | Swap70 | Pkib |
| Rpl23 | Npm1 | Cd79a | Hsp90ab1 | Ifi35 |
| Rpl34 | Bri3 | Snx2 | Sub1 | Rnf213 |
| Vps37b | Kras | Siglecg | Tagap | Samd9l |
| Rpl39 | Srsf2 | Fcer2a | Nfkbiz | Clec2d |
| Rps13 | Rpl38 | Ciita | Hspa8 | Sp100 |
| Txnip | Irs2 | RhobArjgdob | Hilpda | Samhd1 |
| Rps16 | Ets1 | Gimap1 | Dusp2 | Mndal |
| Rps28 | H2-Eb1 | Fcmr | Cd69 | Pml |
| Rpl39 | Hsp90aa1 | Igkc | Samsn1 | Cybb |
| Rps13 | Hnrnpa1 | Actr3 | Eif4a1 | Zufsp |
| Txnip | Nop10 | Ralgps2 | Tgif1 | H2-T23 |
| Rps16 | Dusp2 | AW112010 | Nfkb1 | Helz2 |
| Rps28 | Kdm6b | Ighd | Gch1 | Smchd1 |
| Rpl32 | Hmgb2 | Hsph1 | Irs2 | Psmb9 |
| Rps10 | Odc1 | Gem | Nfkbie | Irf1 |
| Rpl12 | Cacybp | Gm8369 | Npm1 | Tap1 |
| Rpl3 | Ppp1r16b | Psmb9 | Hsp90aa1 | Psme1 |
| Rps3 | Stk17b | Rnase6 | Zc3h12a | Plac8 |
| Rps11 | Rps13 | Lsp1 | Traf4 | Sp110 |
| Mef2c | C1qbp | Fchsd3 | Eif1 | H2-T22 |
| Rpl7 | Rps27 | Apobec3 | Nolc1 | Gm8369 |
| Rpl35 | Rpl8 | Psmb8 | Timm8a1 | Tapbp |
| Rps18 | Tra2b | Actb | Nfatc1 | Ctss |
| Rps27a | Gnl3 | Ca[g | Alkbh1 | Cd47 |
| **FC5** | **FC6** | **FC7** | **FC8** | **FC9** |
| Lyz2 | Gm42418 | Cyb5a | Csf2rb2 | Fabp4 |

| Lgals3 | mt-Co2 | Lgals1 | Csf2rb | Ly6c1 |
| --- | --- | --- | --- | --- |
| Cd14 | mt-Cytb | Pafah1b3 | Fcer1a | Igfbp7 |
| S100a4 | mt-Co3 | mt-C01 | Csf1 | Aqp1 |
| Cxcl2 | mt-Co1 | Ahnak | Ms4a2 | Gng11 |
| Fcer1g | mt-Atp6 | Igkc | Spry2 | Cav1 |
| S100a6 | mt-Nd2 | Ifitm3 | Slpi | Ligp1 |
| Mt1 | mt-Nd4 | Jun | Fcgr3 | Tm4sf1 |
| Ctsb | mt-Nd1 | S100a6 | Hgf | Gpihbp1 |
| Il1b | mt-Nd3 | mt-Atp6 | Serpine2 | Sparc |
| C1qb | Ybx1 | Ptms | Mboat1 | Egfl7 |
| Ifitm3 | G3bp1 | Zeb2 | Nlrp3 | Cxcl12 |
| C1qa | Tmed10 | Id3 | Gsr | Flt1 |
| Thbs1 | A630001G21Rik | Rhob | Emilin2 | Cldn5 |
| Ifitm2 | Scd1 | Cst3 | Sytl3 | Tcim |
| Hmox1 | Sdf4 | mt-Co3 | Slc18a2 | Sparcl1 |
| Cd44 | Taf1d | Cdkn2d | Itga2b | Rgs5 |
| Ccl6 | Hnrnpc | mt-Nd4 | Csrp3 | Cdh5 |
| Ccl9 | Ube2b | Vpreb3 | Klf5 | Cavin2 |
| Selenop | Chmp2a | Lmna | Il18rap | Timp3 |
| Anxa2 | Drap1 | Gadd45g | Itk | Ctla2a |
| Ahnak | Ywhae | Nfkbia | Aqp9 | Timp4 |
| Tgfbi | Med28 | Lmo4 | Slco2b1 | Adgrf5 |
| Tmem176a | Gnb1 | Jund | Il18r1 | Cavin1 |
| Mafb | Pdcd4 | Gadd45b | Il15 | Epas1 |
| C1qc | Ptges3 | Vim | Cdh1 | Slc9a3r2 |
| Tmem176b | Psmd11 | Ly6d | Rab44 | Tcf15 |
| Grn | Actr3 | mt-Co2 | Npl | Cd300lg |
| Alox5ap | Aimp1 | mt-Cytb | Cd7 | Lpl |
| Mgl2 | Sf3b6 | Fth1 | Hcar2 | Mgll |
| Aif1 | Capza2 | Btg2 | Tbc1d4 | Apold1 |
| Lst1 | Fam32a | Dnajc7 | Padi2 | Tspan7 |
| Cdkn1a | Tpm3-rs7 | Hspa1a | Nkg7 | C1qtnf9 |
| Ms4a6c | Pkig | Nr4a1 | Adora2b | Ptprb |
| Basp1 | Morf4l1 | Gpx4 | Alox5 | Cav2 |
| Ccl2 | Swap70 | Itm2b | Alox15 | Ramp2 |
| Plin2 | Ypel3 | Gm42418 | Lag3 | Kdr |
| Tnip3 | Gpr171 | Hmgb2 | Perp | Esam |
| Mrc1 | Atpif1 | Hspa8 | Ms4a3 | Kitl |
| Ptafr | Vamp8 | Rbm38 | Adgrg3 | Id1 |
| Il1rn | Arhgdia | Vgll4 | Cd200r4 | Sox17 |
| Arl4c | Eef2 | Fosb | P2rx1 | Cdkn1c |
| Emp1 | Top1 | Hspa1b | Ikzf2 | Cxcl9 |
| Ccr2 | Gpx4 | Serinc3 | Rapsn | Ehd2 |
| Metrnl | Anp32b | Serpinb1a | Cst7 | Adgrl4 |
| Cxcl16 | Arhgef1 | mt-Nd1 | Fcer1g | Jam2 |
| Lilr4b | Csde1 | Irf1 | Igsf6 | Adamts1 |
| Mpeg1 | Ube2d2a | Spib | Nedd4 | Col4a2 |
| ler3 | Eif3c | Iglc1 | Fyb | Aqp7 |
| Cd300c2 | Blnk | Gngt2 | Gm20186 | Col4a1 |

| **FC10** |
| --- |
|  |
| Retnlg |
| Ngp |
| Lcn2 |
| Wfdc21 |
| G0s2 |
| Ifitm6 |
| Ltf |
| Hp |
| Mmp8 |
| Trem1 |
| Mmp9 |
| Stfa2l1 |
| Mcemp1 |
| Slfn4 |
| Asprv1 |
| Mgst1 |
| Cd177 |
| Mrgpra2b |
| Ly6g |
| Lrg1 |
| Fpr1 |
| A530064D06Rik |
| Mapk13 |
| Trem3 |
| Csf3r |
| Pilra |
| Chil1 |
| Fpr2 |
| Padi4 |
| Krt83 |
| Il1f9 |
| AC110211.1 |
| Itgb2l |
| 9830107B12Rik |
| Ceacam10 |
| Mrgpra2a |
| Tmem40 |
| Crispld2 |
| Dhrs9 |
| F5 |
| Upp1 |
| Ppp1r42 |
| Gm38843 |
| Gpr27 |
| Ankrd22 |
| Chil5 |
| Gm1604a |
| C3 |
| Sgms2 |
| Pilrb2 |
