# Supplementary material for: A Population of M2 Macrophages Associated With Bone Formation
Source: Front Immunol. 2021 Oct 12;12:686769. doi: 10.3389/fimmu.2021.686769 (PMC8547272; doi:10.3389/fimmu.2021.686769)
Supplement: Supplementary Table 2 — Macrophage markers (MH0-MH6) in FC5. The transcripts from FC5 were compared to the top 50 transcripts of each cluster MH0 to MH7. A plus sign indicates the presence of a corresponding transcript in FC5, while a minus sign indicates the absence of a corresponding FC5 transcript. [file Table_2.docx]

**Supplemental Table 2. Macrophage markers (MH0-MH7) in FC5**

| MH0 | FC5 | MH1 | FC5 | MH2 | FC5 | MH3 | FC5 |
| --- | --- | --- | --- | --- | --- | --- | --- |
|  |  |  |  |  |  |  |  |
| Sepp1 | - | Rps29 | - | Folr2 | + | H2-Aa | + |
| Mif1 | - | Rps27 | + | Wfdc17 | + | H2-Eb1 | + |
| Pf4 | + | Rpl37a | - | Sepp1 | + | Cd74 | + |
| Gapdh | + | Rpl35a | - | Ccl6 | + | H2-Ab1 | + |
| Pdpn | - | Rps14 | - | Fcgrt | + | Tmem176b | - |
| Pkm | + | Rpl13a | - | Ednrb | - | Tmem176a | - |
| Lgals1 | + | Capg | - | Ltc4s | + | H2-DMb1 | + |
| Aldoa | - | Lgals3 | + | Fcna | - | H2-DMa | + |
| S100a4 | + | Lgals1 | + | Thbs1 | + | Ctsh | - |
| Arg1 | - | Anxa2 | + | Cbr2 | + | Lgals1 | + |
| S100a6 | + | Ppia | - | Clec10a | + | H2-D1 | + |
| Tpi1 | + | S100a4 | + | Tslp | - | Apoe | + |
| Ifitm3 | + | Prdx1 | + | Timp2 | + | Plbd1 | + |
| Pltp | + | S100a6 | + | Atf3 | + | Trf | + |
| Eno1 | + | Rps9 | - | Lyve1 | - | Ubb | -_ |
| Ly6e | - | Calm1 | + | Rcn3 | - | Cd83 | - |
| Bnip3 | - | Pfn1 | + | Gas6 | - | Pla2g7 | + |
| Pgam1 | + | B2m | + | Igf1 | - | Il1b | + |
| Vegfa | - | Cfl1 | + | Cxcl16 | + | Arl5c | + |
| Capg | - | Ctls | - | Pltp | + | Ifitm2 | + |
| Jund | - | S100a10 | + | Ifitm3 | + | Srgn | + |
| Ldha | - | S100a11 | + | Serpinb6a | - | Lyz2 | + |
| Anxa2 | + | Ldha | - | Id2 | + | Cst3 | + |
| Fn1 | + | H2-D1 | + | F13a1 | + | Spp1 | - |
| Clec4d | + | Clic1 | + | Pepd | - | Syngr1 | - |
| Basp1 | + | Gapdh | + | Aldh2 | - | Cyp4f18 | - |
| Tmem176b | + | Eno1 | + | Ccl8 | - | Cd52 | - |
| Serinc3 | - | Cd68 | + | Clec4d | + | Mif | - |
| Itm2b | + | Ms4a6d | + | Fth1 | + | Pf4 | + |
| Wfdc17 | + | Ctss | + | Ap2m1 | + | Pltp | + |
| Clec10a | + | Atp5b | - | Mt1 | + | Hspa8 | + |
| Lat2 | + | Pkm | + | Ccl2 | + | Pdpn | - |
| Slc16a3 | + | Tmsb10 | - | Selenbp1 | - | Ndufa2 | - |
| Adam8 | + | Rps12 | - | Atox1 | + | Atp5e | - |
| Ccl6 | + | Rps21 | - | Cd52 | - | Unc93b1 | + |
| Trf | + | Jund | - | Basp1 | + | Uqcr11 | + |
| Mxi1 | + | Myl12a | + | Btg1 | + | Ly86 | + |
| Tmem176a | + | Ubb | - | Vegfa | - | Serpinb6a | - |
| Pfn1 | + | Uqcrb | - | Fxyd2 | - | Mrc1 | + |
| Id2 | + | Tpi1 | + | Ly6e | - | Cela1 | - |
| Ctla2a | - | Myl6 | + | Snx5 | - | Cd14 | + |
| Fxyd5 | + | Pgam1 | + | Ccl9 | + | F13a1 | + |
| Aprt | + | Tspo | + | Pmp22 | - | Timp2 | + |
| Igf1 | - | Tagln2 | + | Zfand5 | + | Snx3 | + |
| Cd74 | + | Arpc3 | - | Klf2 | - | Ctsc | + |
| Thbs1 | + | Emp3 | + | Gda | + | Gadd45b | + |
| S100a11 | + | Fxyd5 | + | Hint1 | - | Ifrd1 | + |
| Pgk1 | + | Cotl1 | + | Cd302 | + | Cxcl16 | + |
| Unc93b1 | + | Cbr2 | - | Pfkb3 | - | Gapdh | + |

| MH4 | FC5 | MH5 | FC5 | MH6 | FC5 | MH7 | FC5 |
| --- | --- | --- | --- | --- | --- | --- | --- |
|  |  |  |  |  |  |  |  |
| Ifit3 | **-** | Slc40a1 | **-** | 2810417H13Rik | - | Col15a1 | - |
| Ifit2 | **-** | Gclm | **-** | Nusap1 | - | Tmem47 | - |
| Isg15 | **-** | Ftl1 | + | Birc5 | - | Clec14a | - |
| Ms4a4c | + | Slc48a1 | + | Pbk | - | Abl3bp | - |
| Irf7 | **-** | Fth1 | + | Top2a | - | Erg | - |
| Rsad2 | **-** | Hmox1 | + | Cdca3 | - | Cyr61 | - 9 |
| Zbp1 | **-** | Gm10116 | + | Ccna2 | - | Plpp3 | - 9 |
| Pydc4 | **-** | Prdx1 | + | Lockd | - | Hspg2 | - 9 |
| Ifit1 | **-** | Creg1 | + | Cdkn3 | - | Nfib | - 9 |
| Ifitm3 | + | Gsr | **-** | Prc1 | - | Cd200 | - 9 |
| Rtp4 | - | Srxn1 | **-** | Tk1 | - | Wwtr1 | - 9 |
| Gm4955 | - | Clec4n | **-** | Ube2c | - | Timp3 | - 9 |
| Phf11b | - | Lipa | + | Fam64a | - | Sparcl1 | - 9 |
| Phf11d | - | Slc7a11 | - | Racgap1 | - | Ablim3 | - 9 |
| Oas3 | - | Slc11a1 | + | Ccnb2 | - | Nid1 | - 9 |
| Ly6a | - | Pgd | + | Rrm2 | - | Mecom | - 9 |
| Ube2l6 | - | Blvrb | - | Spc24 | - | Trp53i11 | - |
| Pyhin1 | - | Htatip2 | - | Cdk1 | - | Col4a2 | - 9 |
| Xaf1 | - | Clec4d | + | Cdca8 | - | Mmrn2 | - 9 |
| Mnda | - | Mgst1 | - | Ccnb1 | - | Tm4sf1 | - 9 |
| Ly6e | - | Cyb5a | + | Stmn1 | - | Spint2 | - |
| Bst2 | + | Gstm1 | - | Cks1b | **-** | Ramp2 | - 9 |
| Plac8 | + | Ctsd | - | Hist1h2ap | **-** | Npdc1 | - 9 |
| Oasl2 | - | Txnrd1 | + | Tpx2 | **-** | Tinagl1 | - 9 |
| Irgm1 | - | Cd36 | - | Cenpf | **-** | Sdc2 | - |
| Ifi204 | + | Cat | - | Cenpm | **-** | Fbln2 | - 9 |
| Fcgr1 | + | Gclc | - | Uhrf1 | **-** | Lpar4 | - |
| Mndal | - | Txn1 | + | Mad2l1 | **-** | Emnc | - |
| Parp14 | + | Ednrb | - | Cenpw | **-** | Sdpr | - |
| Ifi47 | - | Ninj1 | + | Knstrn | **-** | Fxyd6 | - |
| Tor3a | - | Sqstm1 | + | Smc2 | **-** | Lims2 | - 9 |
| Ifi203 | - | Akr1b8 | - | Tyms | **-** | Gpihbp1 | - 9 |
| Sp110 | - | Pf4 | + | Mki67 | **-** | Apbb2 | - 9 |
| Trim30a | + | Taldo1 | + | Tmpo | **-** | Cldn5 | - 9 |
| Ifi27l2a | - | Igf1 | - | Spc25 | **-** | Fermt2 | - 9 |
| Oas1a | - | G6pdx | - | Asf1b | **-** | Cd34 | - 9 |
| Fcgr4 | + | Pla2g7 | + | Mcm5 | **-** | Jam2 | - 9 |
| Ifi35 | - | Msrb1 | + | H2afx | **-** | Tmem158 | - |
| Stat1 | - | Abcc1 | - | Ezh2 | **-** | Nedd4 | - 9 |
| Slfn4 | - | Tnfaip2 | + | Lsm2 | **-** | Emp2 | - |
| Psmb8 | - | Gdf15 | - | Smc4 | **-** | Igfbp7 | - 9 |
| Tap1 | - | Aldoa | - | Gmnn | **-** | Cdh5 | - 9 |
| H2-T22 | - | Por | + | Tacc3 | **-** | Bcam | - 9 |
| Ifih1 | - | Akr1a1 | + | Tuba1b | + | Ptrf | - |
| B2m | + | Pdlim4 | - | Cks2 | + | Kdr | - 9 |
| Sp100 | - | Gde1 | - | Ptma | - | Ly6c1 | - 9 |
| Rnf213 | - | Adam8 | + | Hmgn2 | - | Cttn | - |
| Lgals3bp | + | Prdx6 | - | H2afz | + | Fxyd1 | - |
| Pnp | + | S100a4 | + | Nucks | - | Cdc42ep1 | - |
| Dck | - | Cstb | + | Hmgb1 | - | Amotl2 | - 9 |
